# Deep Learning for Chest X-ray Diagnosis: Competition Between Radiologists with or Without Artificial Intelligence Assistance

**DOI:** 10.1007/s10278-024-00990-6

**Published:** 2024-02-08

**Authors:** Lili Guo, Changsheng Zhou, Jingxu Xu, Chencui Huang, Yizhou Yu, Guangming Lu

**Affiliations:** 1 Department of Radiology, The Affiliated Huaian No. 1 People’s Hospital of Nanjing Medical University, Huai’an, 223300 China; 2 Department of Medical Imaging, Jinling Hospital, Medical School of Nanjing University, Nanjing, 210002 China; 3 Deepwise AI Lab, Beijing Deepwise & League of PHD Technology Co., Ltd, Beijing, 100080 China

**Keywords:** Chest X-ray, Artificial intelligence, Competition, Deep learning, Score

## Abstract

This study aimed to assess the performance of a deep learning algorithm in helping radiologist achieve improved efficiency and accuracy in chest radiograph diagnosis. We adopted a deep learning algorithm to concurrently detect the presence of normal findings and 13 different abnormalities in chest radiographs and evaluated its performance in assisting radiologists. Each competing radiologist had to determine the presence or absence of these signs based on the label provided by the AI. The 100 radiographs were randomly divided into two sets for evaluation: one without AI assistance (control group) and one with AI assistance (test group). The accuracy, false-positive rate, false-negative rate, and analysis time of 111 radiologists (29 senior, 32 intermediate, and 50 junior) were evaluated. A radiologist was given an initial score of 14 points for each image read, with 1 point deducted for an incorrect answer and 0 points given for a correct answer. The final score for each doctor was automatically calculated by the backend calculator. We calculated the mean scores of each radiologist in the two groups (the control group and the test group) and calculated the mean scores to evaluate the performance of the radiologists with and without AI assistance. The average score of the 111 radiologists was 597 (587–605) in the control group and 619 (612–626) in the test group (*P* < 0.001). The time spent by the 111 radiologists on the control and test groups was 3279 (2972–3941) and 1926 (1710–2432) s, respectively (*P* < 0.001). The performance of the 111 radiologists in the two groups was evaluated by the area under the receiver operating characteristic curve (AUC). The radiologists showed better performance on the test group of radiographs in terms of normal findings, pulmonary fibrosis, heart shadow enlargement, mass, pleural effusion, and pulmonary consolidation recognition, with AUCs of 1.0, 0.950, 0.991, 1.0, 0.993, and 0.982, respectively. The radiologists alone showed better performance in aortic calcification (0.993), calcification (0.933), cavity (0.963), nodule (0.923), pleural thickening (0.957), and rib fracture (0.987) recognition. This competition verified the positive effects of deep learning methods in assisting radiologists in interpreting chest X-rays. AI assistance can help to improve both the efficacy and efficiency of radiologists.

**Supplementary Information:**

The online version contains supplementary material available at 10.1007/s10278-024-00990-6.

## Introduction

In China, radiologists, especially young doctors, have a heavy workload of reading plain X-ray films every day. Diagnostic radiologists have to face the burdensome task of reviewing these images, which occupies much of their time and attention [1]. Nevertheless, the diagnosis of chest films requires experience and time. Reducing subjective human errors and improving efficiency have long been goals that everyone in this field would like to achieve. Chest radiography is the most common type of imaging examination in the world, with over 2 billion procedures performed each year [2]. This technique is critical for the screening, diagnosis, and management of thoracic diseases, many of which are among the leading causes of mortality worldwide. Chest radiography is a very common examination, and X-ray imaging is the primary diagnostic tool used by radiologists to preliminary assess patients for chest diseases. Missing a lesion in a radiograph often has severe consequences for patients, resulting in delayed further examination and treatment. A computer system to interpret chest radiographs as effectively as practising radiologists could thus provide substantial benefit in many clinical settings, from improved workflow prioritization and clinical decision-making support to large-scale screening and global population health initiatives.

Artificial intelligence (AI), particularly deep learning, is currently being developed in an effort to assist radiologists. Recently, a deep learning model was found to match expert human radiologists in diagnosing 10 or more pathologies on chest radiographs [3]. Automated diagnosis via chest imaging has received increasing attention, with specialized algorithms developed for pulmonary tuberculosis classification and lung nodule detection, but the use of chest radiographs to discover other pathologies, such as pneumonia and pneumothorax, necessitates an approach that can detect multiple pathologies simultaneously [2]. Only recently has the computational power and availability of large datasets enabled the development of such an approach. The National Institutes of Health’s release of ChestX-ray14 led to many more studies on using deep learning for chest radiograph diagnosis [4, 5]. However, the performance of these algorithms has not been demonstrated in a real clinical situation.

In this work, we aimed to assess the performance of a deep learning algorithm in helping radiologist achieve improved efficiency and accuracy in chest radiograph diagnosis. We adopted a deep learning algorithm to concurrently detect the presence of normal findings and 13 different abnormalities in chest radiographs and evaluated its performance in assisting radiologists.

## Methods

### Data

This study is based on a chest X-ray image database of 4098 patients admitted to our hospital. For this experiment, the engineer of the Department of Medical Imaging retrieved the image data from April 2007 to June 2019 in the picture archiving and communication system (PACS) and the corresponding clinical information data in the radiology information system (RIS). The image inclusion criteria were as follows: 1) complete clinical and imaging data, 2) clear and complete images without motion artifacts or metal artifacts affecting observation, and 3) contemporaneous chest CT and frontal plain radiograph images. In order to ensure that there was no bias in the observation of the lesions, the interval between the two examinations was limited to 1 day (24 h). The original pathology labels were organized from the clinical CT reports. A set of 100 clear frontal chest radiographs was selected from the database for the competition according to the following criteria [6]: (1) ability to distinguish hilar shadow structures; (2) ability to distinguish lung textures in clavicle, mammary gland, and left heart shadows; (3) full visualizability of the tips of the lungs; (4) projection of the scapula beyond the lung field; (5) bilateral thoracic lock joint symmetry; (6) visualization of the diaphragm with complete and sharp edges; and (7) clear and sharp margin of heart and mediastinum. According to these criteria, two radiologists with more than 10 years of experience jointly reviewed the chest films and assessed their quality; X-ray films that did not meet the criteria were excluded. The set was curated to contain at least 10 cases of each pathology by randomly sampling cases and iteratively updating the selected cases by sampling from the underrepresented pathologies. The anonymization of all data was guaranteed in this research.

The 100 radiographs in the competition, which were independent of the multi-center data used to build the algorithm model, were further annotated by 3 independent board-certified cardiothoracic specialist radiologists (average experience, 20 years; range, 18–28 years) for the presence of normal findings and 13 different abnormalities. The frontal chest radiographs were labeled based on coronal CT reconstructions as the gold standard. There was no patient overlap among the partitions. All experts were permitted to review the patient’s history or prior examinations, and that evaluation was limited to a dataset from a single institution. We invited the experts to the hospital where the data were collected, where they could adjust their diagnoses by referring to the CT images, past images of the patients, and relevant clinical data on the PACS. The data from only a single hospital were used to avoid the interference of different machines and scanning conditions. For the included images, CT images were available for comparison at the same time, and the correct answers were determined after consistent interpretation by multiple experts. This experiment is designed to imitate the real working status of radiologists with diagnostic X-rays and to determine how AI can help doctors. Therefore, we randomly divided the 100 radiographs into a control group, consisting of the first 50 selected radiographs, which was assessed independently by the physicians without the help of AI, and a test group, consisting of the remaining 50 radiographs, which was assessed by the physicians with AI assistance of AI. The experts who were responsible for setting the gold standard performed a comparative analysis of the image data between the control group and experimental group and attempted to ensure that the number of each of the 14 pathologies in the X-ray films of the two groups were similar and that the difficulty of diagnosis was similar. There was no significant difference in the number of pathological changes between the two groups (*P* > 0.05).

**Fig. 1:**
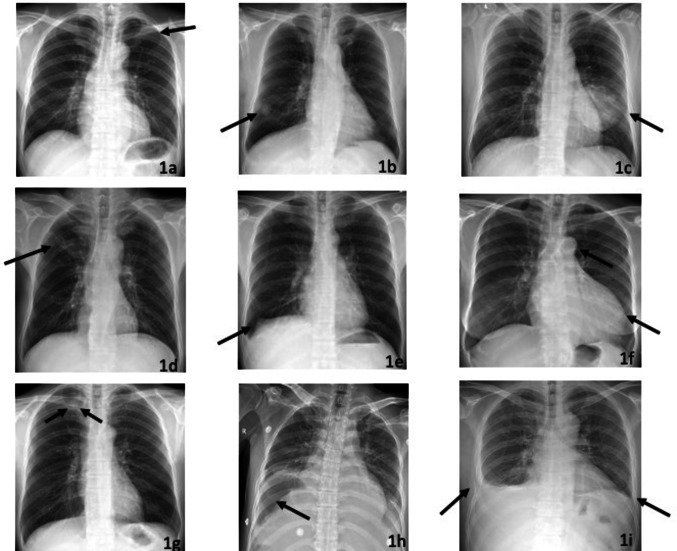
Typical radiographs showing chest abnormalities. **a** A small amount of pneumothorax on the left side. **b** A lung nodule in the right lower lobe. **c** A lung mass in the left lower lobe. **d** A cord shadow in the right upper lung lobe. **e** Thickening of the right pleura. **f** Aortic calcification and an enlarged heart shadow. **g** A cavity shadow in the right upper lung lobe. **h** Free air under the right diaphragm. **i** Fracture of the 7th rib on the right, with pleural effusion on both sides

**Fig. 2:**
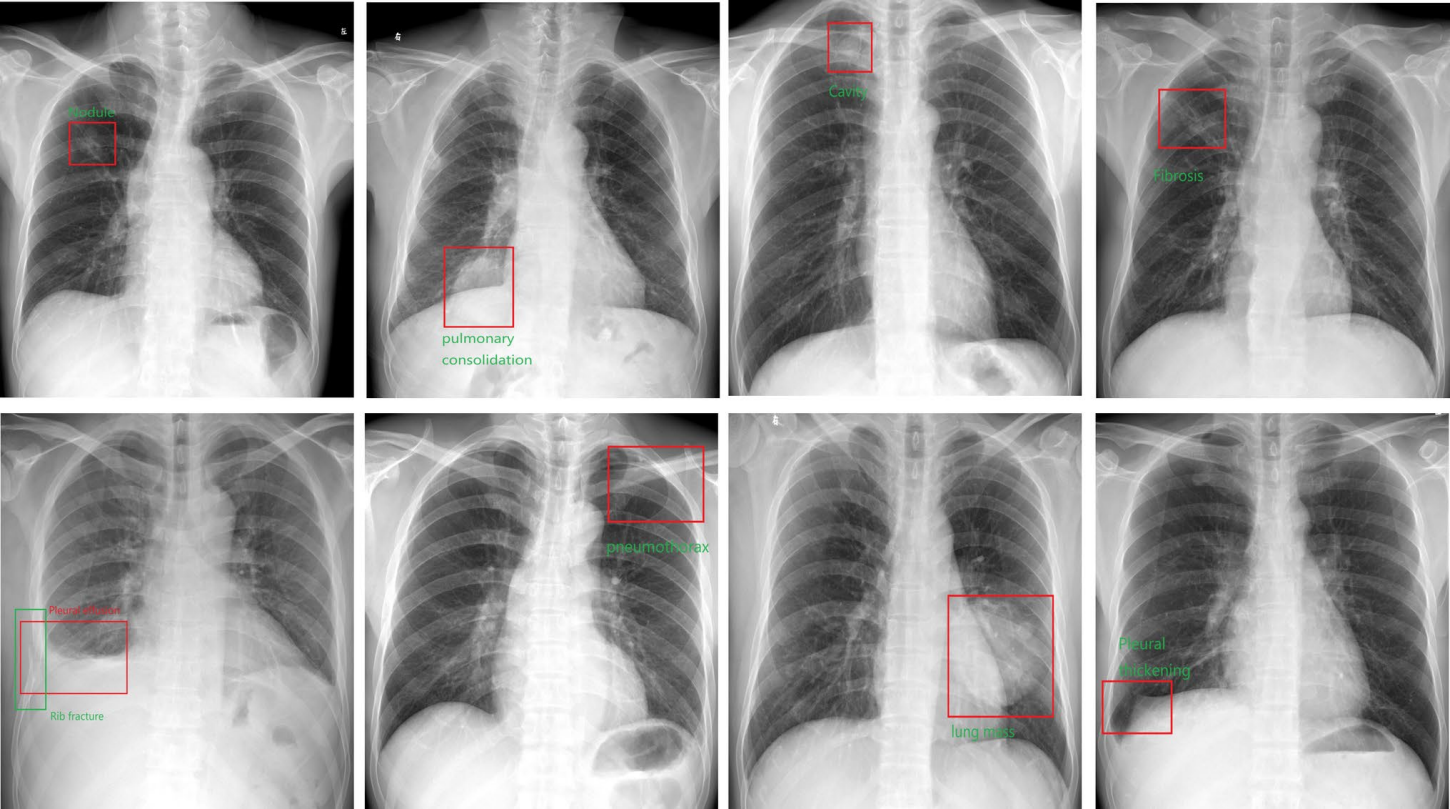
The AI system output the bounding boxes and labels of the lesions to assist radiologists

Typical abnormalities are shown in Fig. 1 and the AI system output the bounding boxes and labels of the lesions to assist radiologists (Fig. 2).

### Computer-Aided Diagnosis System

The experiment was conducted using the Dr. Wise system, an intelligent imaging system, which was used to store the original DICOM image data in the cloud for later retrieval, view, and intelligent auxiliary diagnostic operation, and which was integrated with the chest X-ray AI model as part of its function.

**Fig. 3:**
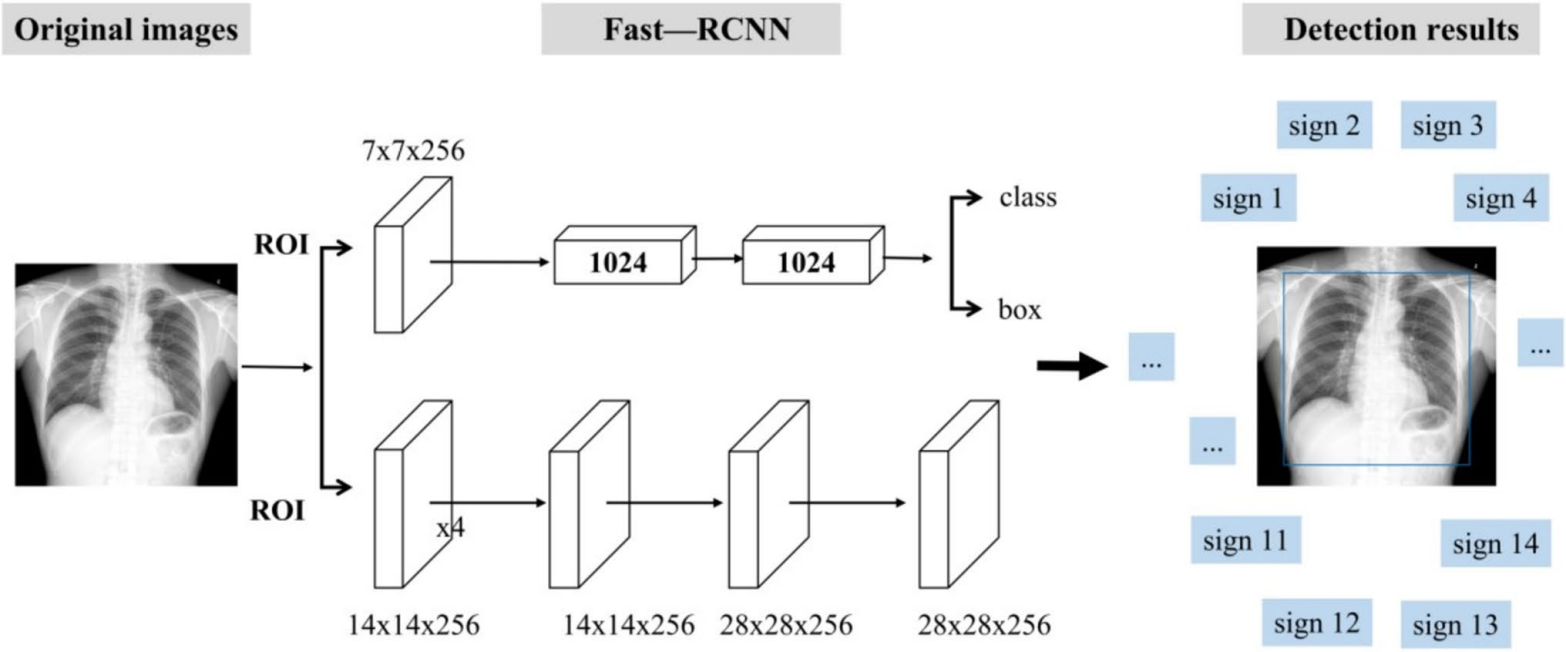
Flowchart of this study. Note: one hundred chest radiographs were randomly divided into two groups at a 1:1 ratio. Images in the control group were independently marked by 111 radiologists, and those in the test group were labeled by radiologists with AI assistance. Each radiologist interpreted the presence of the 14 signs in each case. The total score was recorded, and the scores of the radiologists without and with AI assistance were compared in detail

The chest X-ray AI model can detect normal chest X-rays and those of 13 different pathologies, including fibrosis, heart shadow enlargement, masses, pleural effusions, pulmonary consolidation, aortic calcification, calcification, cavities, nodules, pleural thickening, rib fractures, subphrenic free air, and pneumothorax. The AI chest X-ray algorithm used in this experiment was published in a related technical paper [7], in which the training data were further expanded to more than 30,000 images. The current AI model (Fig. 3) in the Dr. Wise system uses a large amount of data, improving the model performance and generalization.

### Details of Algorithm Development

We used Faster R-CNN [8], a high-performance convolutional neural network for object detection, to output the category and the location (bounding box) of the lesion [7]. Faster R-CNN is supported by the feature pyramid network, which excels at identifying lesions on various scales, e.g., from nodules to consolidation. The first stage is the region proposal network (RPN) [9], which outputs nearly 2000 regions of candidate lesions. The second stage is composed of region of interest (ROI) pooling and head layers. For lesion recognition and localization, there are two heads of fully connected layers for classification and bounding box regression, respectively. Finally, non-maximum suppression is adopted to remove redundant boxes and output the final results as a reference for doctors.

More than 30,000 chest X-ray images were collected from multiple medical institutions to build the algorithm model. The training procedure was implemented using PyTorch^1^ on 4 TITAN-V GPUs. ResNet-50 [10] was pretrained on ImageNet. We applied stochastic gradient descent (SGD) with a weight decay of 0.0001 and momentum of 0.9 for optimization. The first conversion layers of feature pyramid networks for object detection (FPN) and C4 were frozen. We trained 50 epochs with an image batch size of 2 on each GPU. The learning rate started at 0.01 and was reduced by a factor of 10 after 20 and 40 epochs. During training, we adopted random flipping and multiscale sampling for all images. At the testing stage, the shorter side of the image was fixed at 1200 pixels. The performance of the AI model on internal datasets is detailed in Supplement 1. Different from most existing works that have used class activation mapping (CAM) [11] to visualize possible lesion areas, our network directly provided categories and bounding boxes for the predicted lesions, which was more accurate and helpful for doctors.

### Radiologist Competition

**Fig. 4:**
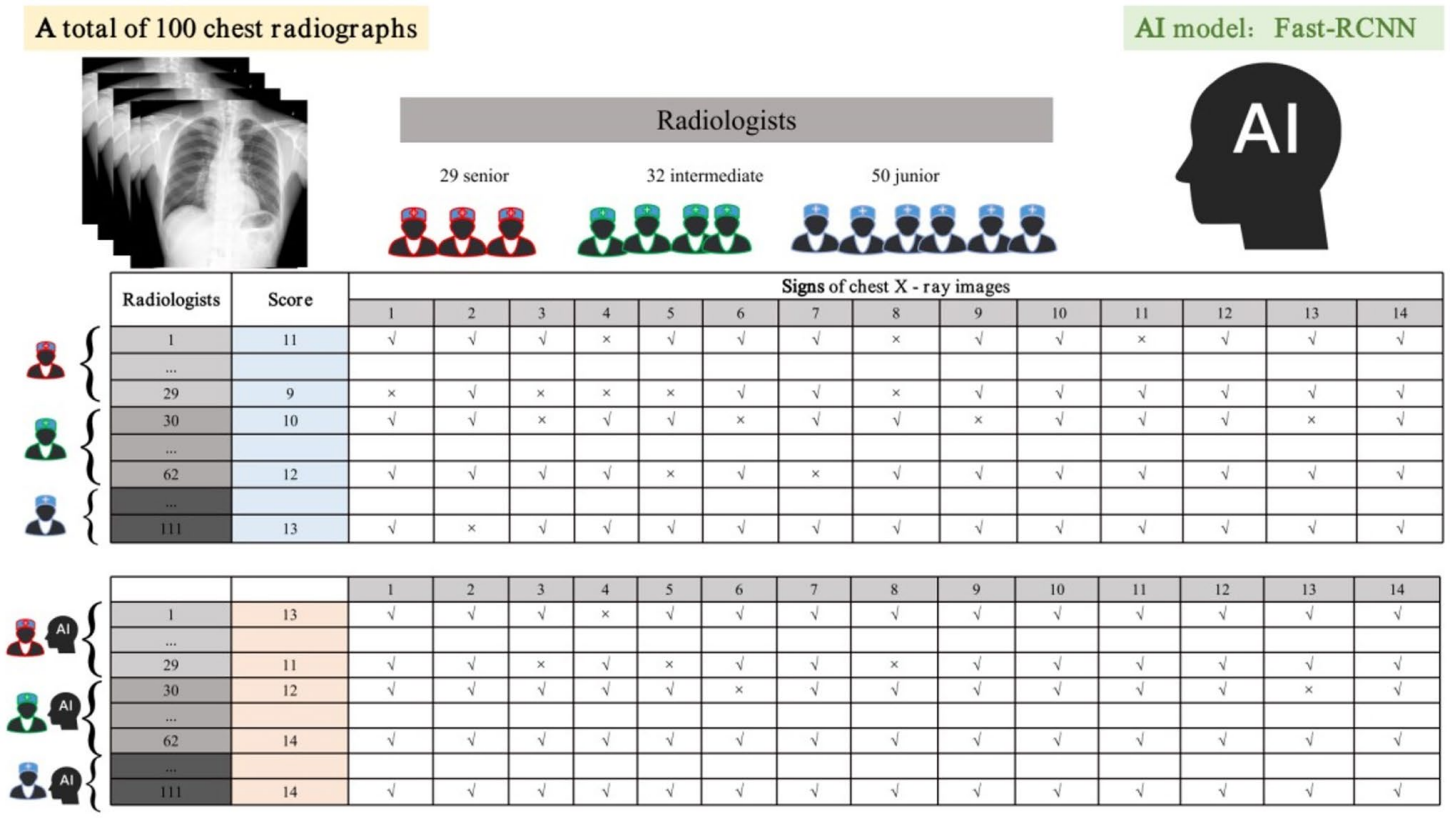
Fast–R-CNN detection algorithm

The flowchart of this study is shown in Fig. 4. To verify the performance of the AI algorithm in assisting radiologists in diagnosis, 111 radiologists participated in the competition and observed 50 radiographs without and with AI assistance for all 14 labels. We randomly split the data of 100 cases into two groups at a ratio of 1:1. The data of the 50 cases in the control group were independently labeled by the radiologists alone, and the data of the 50 cases in the test group were labeled by the radiologists with AI assistance.

In this competition, a university computer teaching classroom with 120 available computers was used. Before the competition, the computers were all installed with the Dr. Wise system. Cloud PACS was used for storage and sharing of image data, and cloud AI-assisted diagnosis was used to automatically retrieve cloud image data for computer vision-based artificial intelligence processing to detect and segment lesions. The reorganization and other results are presented in the cloud RIS for doctors to consult; the cloud PACS is used for doctors to assign the lesion labels. For each image, each sign was labeled “no” or “yes”; each image was allowed to have multiple signs. The reading environment and computer settings were the same for all participants in the competition.

We recruited radiologists in a provincial annual radiologist meeting, explained the purpose and process of the competition and the participant requirements for recruitment, and sent a link to a webpage where the radiologists could fill in the registration information. A total of 128 radiologists entered the competition. After reviewing their background information, we persuaded 6 doctors from the hospital that provided the image data (involving data confidentiality) and 11 postgraduate students majoring in medical imaging (because of insufficient working years) to withdraw from the competition. Among the 111 participating radiologists, 50 were junior radiologists (less than 6 years of working experience), 32 were intermediate radiologists (6–14 years of working experience), and 29 were senior radiologists (at least 15 years of working experience). The Medical Ethics Review Board of the local hospital approved this study, and all radiologists consented to participate in the labeling process. All radiologists individually reviewed and labeled each of the images using a freely available image viewer with capabilities for picture archiving and communication system features, such as zoom, window leveling, and contrast adjustment. The radiologists could have access to simple patient information or the disease prevalence in the data. Labels were entered into a standardized data entry program, and the time to complete each individual review and all reviews together were recorded.

### Scoring Rules

Each image in the competition was annotated with 1 normal or up to 13 different abnormal labels, corresponding to the 14 X-ray signs (14 pathological findings). Each competing radiologist had to determine the presence or absence of these signs based on the label provided by the AI. These labels are referred to as the 14 X-ray signs, including signs of bony abnormalities in the ribs, bullae in the lungs, pulmonary infiltrative lesions, atelectasis, masses (> 3 cm), nodules (< 3 cm), fibrosis, calcification, cavities, heart shadow enlargement, aortic calcification, pleural thickening, pleural effusion, pneumothorax, and gas separation downstream of the diaphragm. We used the results of the joint diagnoses from experts with 18 and 28 years of experience combined with CT and X-ray findings as the reference standard. Two groups of data were randomly selected at a 1:1 ratio: the non-AI-assisted group and the AI-assisted group, with 50 cases in each group. A total of 111 radiologists (29, senior; 32, intermediate; and 50, junior) were selected to judge the 14 signs in the two groups of images.

A radiologist was given an initial score of 14 points for each image read, with 1 point deducted for an incorrect answer and 0 points given for a correct answer. Thus, the maximum score for each person in each group was 700 points (50 pictures × 14 points per picture = 700 points). The final score for each doctor was automatically calculated by the backend calculator. Finally, we calculated the mean scores of each radiologist in the two groups (the control group and the test group) and calculated the mean scores of each senior, intermediate and junior radiologist in the two groups to evaluate the performance of the radiologists with and without AI assistance.

### Statistical Analysis

All statistical analyses were completed in the R environment for statistical computing. The R package was used to calculate the exact Fleiss kappa and Cohen kappa. The boot package was used to perform the bootstrapping and construct the corresponding 95% confidence intervals (95% CIs). The ConSpline package was used to estimate the receiver operating characteristic (ROC) curve for the radiologists using partial least-squares regression with constrained splines, the pROC package was used to estimate the ROC curve for the algorithm, and the MESS package was used to calculate the area under the ROC curve (AUC) for the radiologists with and without AI assistance. Figures were created using the ggplot2 and gridExtra packages. Data are expressed as the median (25th–75th percentile) (*M* (P25–P75)) and were analyzed using the Wilcoxon signed-rank test. Moreover, we calculated the statistical power of this study, and the final calculated statistical power value was 0.83, which is greater than the general statistical power value (power = 0.8), indicating that our study has sufficient credibility.

## Results

### AI and Radiologists’ Scores in Diagnosing Chest Radiographs

**Fig. 5:**
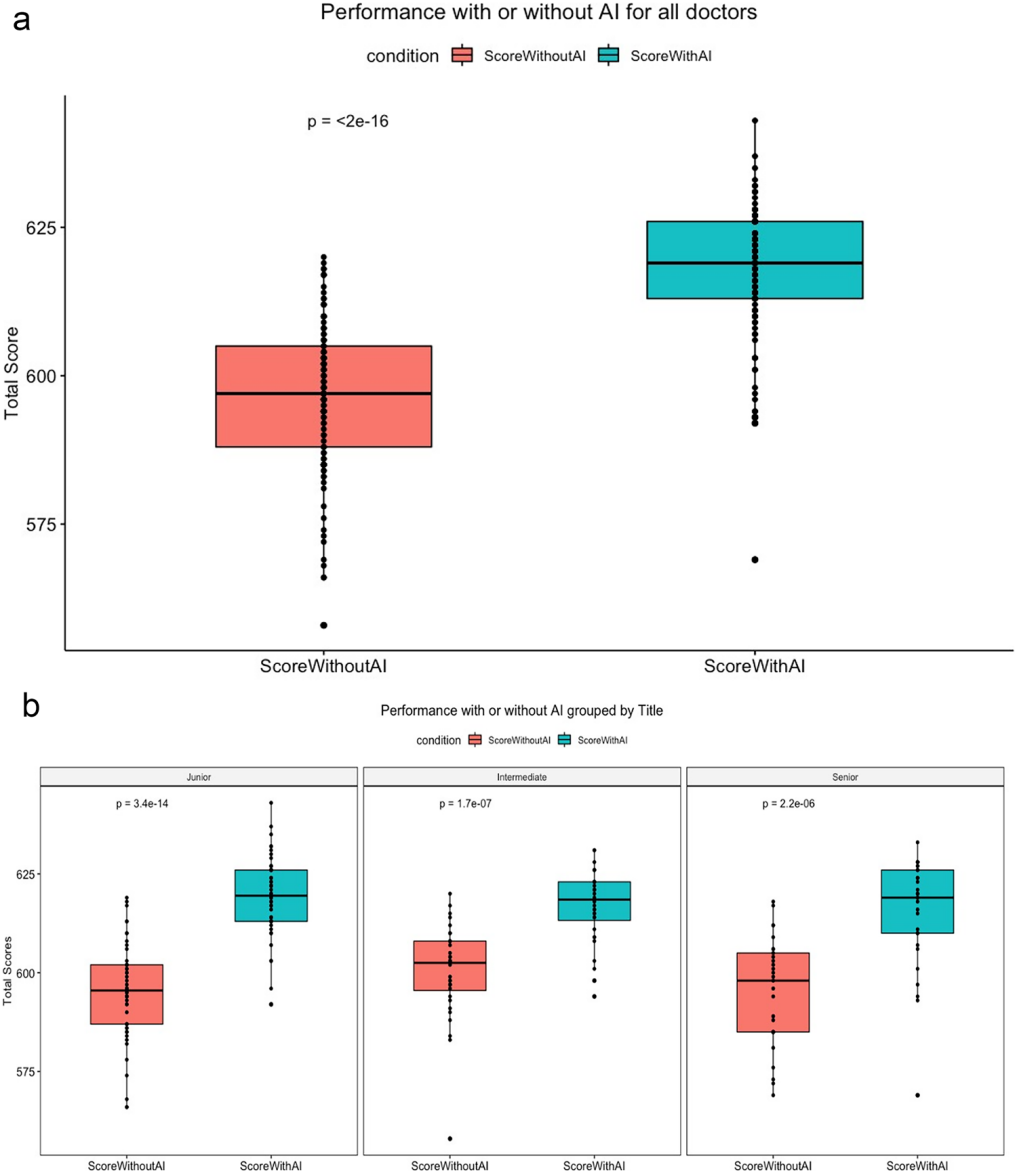
Effects of the relabeling procedure on AI performance. **a** The AI relabeling procedure resulted in an increase in the total score for all radiologists. **b** The mean proportion of correct values with the scores of the junior, intermediate, and senior radiologists are illustrated

All participating radiologists assessed the first 50 chest radiographs without AI assistance and the second 50 chest radiographs with AI assistance. The average score achieved by the 111 radiologists was 597 (587–605) in the first 50 cases and 619 (612–626) in the second 50 cases (*P* < 0.001). Among them, the average scores achieved by the 50 junior doctors were 593 (583.75–595) and 613 (607.75–619.75), respectively (*P* > 0.05). The average scores achieved by the 32 intermediate doctors were 597 (590.5–604.5) and 622 (616.5–626), respectively (*P* < 0.05). The average scores achieved by the 29 senior doctors were 599 (588–606) and 616 (610–623.5), respectively (*P* < 0.05). The performance of all radiologists with and without AI assistance is illustrated in Fig. 5.

**Fig. 6:**
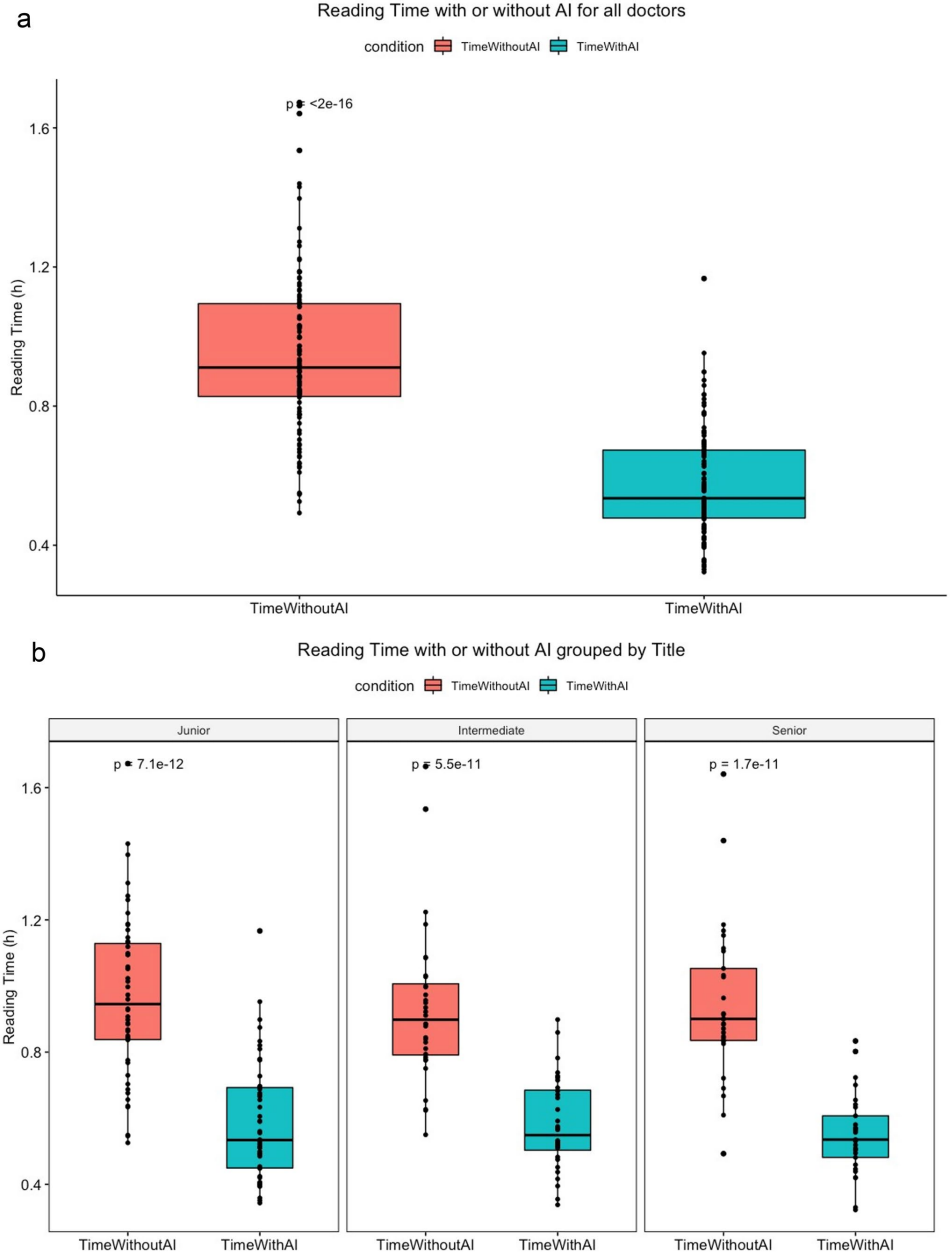
The total time for radiologists to assess the first and second 50 chest radiographs (Fig. 5a). The total time for junior, intermediate, and senior radiologists to assess the first and second 50 chest radiographs (Fig. 5b)

The time spent by 111 radiologists on the first and second 50 cases was 3279 (2972–3941) s and 1926 (1710–2432) s, respectively (*P* < 0.001). The time spent by junior doctors on the first and second 50 cases was 3148 (2730–3793) s and 1771 (1439–2167) s, respectively (*P* < 0.05). The time spent by intermediate doctors on the first and second 50 cases was 3417 (2918–3980) s and 1885 (1613–2432) s, respectively (*P* < 0.001). The time spent by senior doctors on the first and second 50 cases was 3215 (2996–3908) s and 2062 (1845–2500) s, respectively (*P* < 0.001). These data are expressed as the median (25th–75th percentile) (*M* (P25–P75)) and were analyzed using the Wilcoxon signed-rank test. The time for radiologists to assess the first and second 50 chest radiographs is displayed in Fig. 6. The average time for the radiologists to interpret the 100 radiographs was substantially longer for the first part (3279 s) than the second part (1926s).

**Fig. 7:**
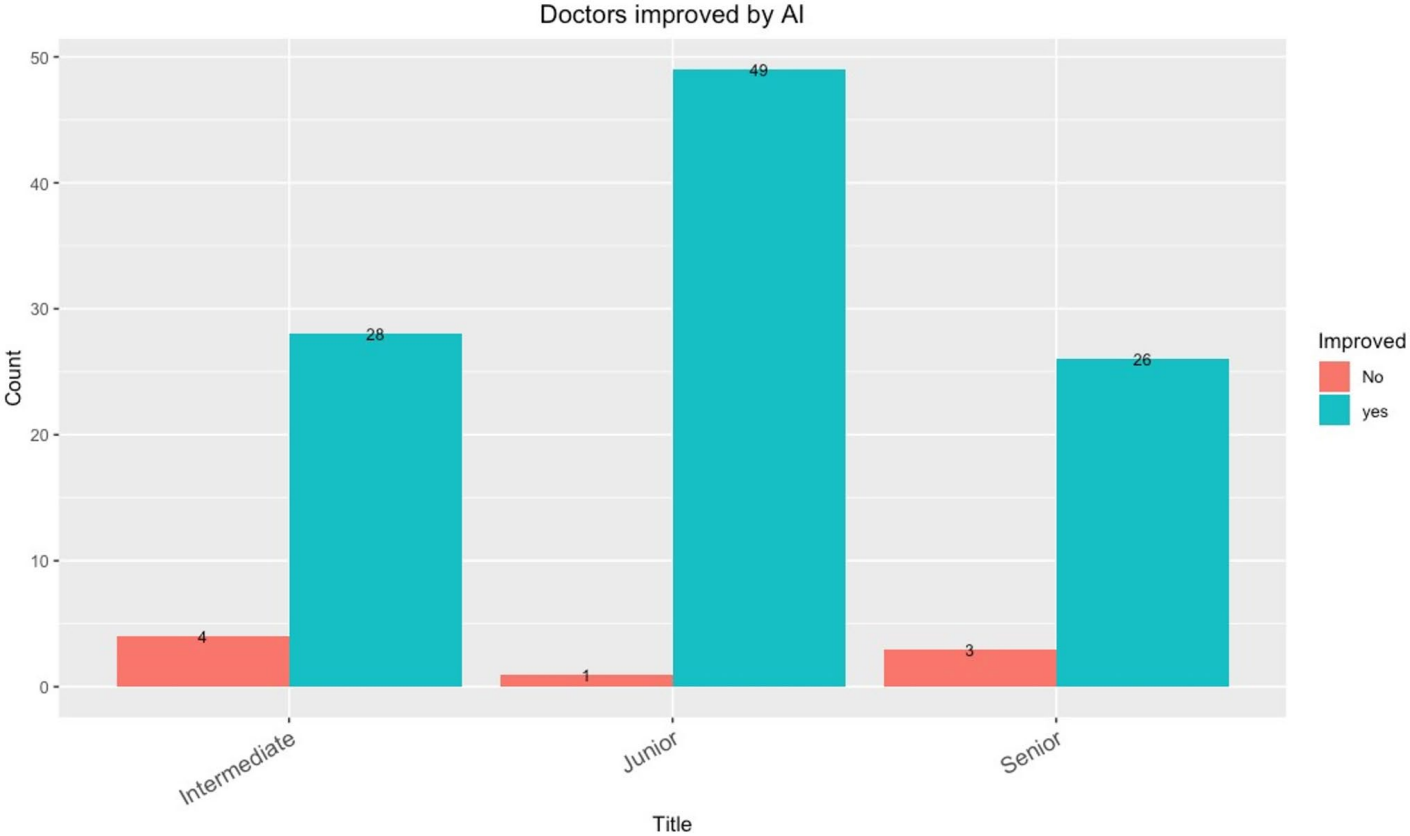
The numbers of junior, intermediate, and senior doctors who achieved improvements in diagnostic accuracy

**Fig. 8:**
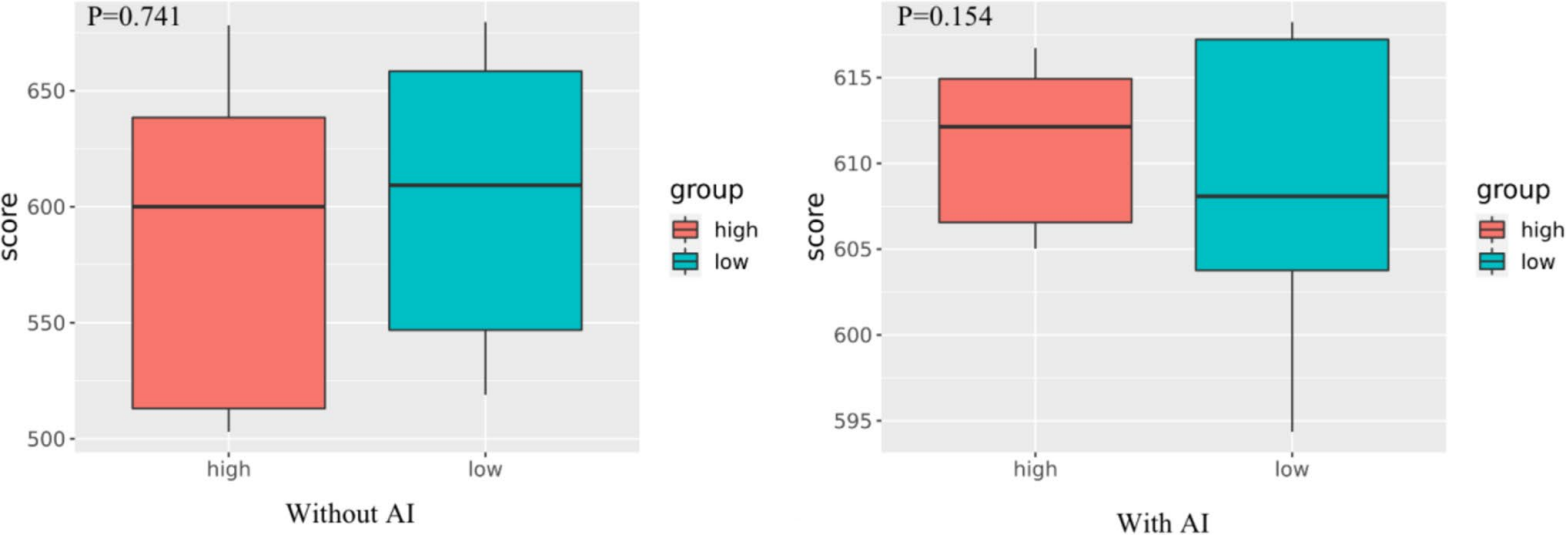
The scores in the high-time-consuming and low-time-consuming groups (with and without AI)

The decrease in the time consumption for all radiologists in the second part showed that AI assistance also helped to improve efficiency. The performance of all radiologists with and without AI assistance showed that AI improved the diagnostic accuracy of the doctors, suggesting that AI can help radiologists provide better healthcare to their patients. The numbers of junior, intermediate and senior doctors who achieved improvements in diagnostic accuracy are shown in Fig. 7. We further analyzed the significance analysis of primary radiologist scores (with and without AI) for different time groups. The results showed that there was no difference in the scores of the group with AI (P=0.741) and the group without AI (P=0.154) between the high and low time consuming groups (Fig 8). The performance of all radiologists in identifying normal findings and 13 abnormalities was different with and without AI assistance. The performance in rib fracture, consolidation, mass, nodule, fibrosis, calcification, cavity, heart shadow enlargement, aortic calcification, pleural thickening, pleural effusion, pneumothorax, and subphrenic free air identification is illustrated, and numerical values for the 14 X-ray signs are reported in Table 1.

**Table 1:** AUC of radiologists and AI with CIs

Fourteen X-ray signs	AI (95% CI)	Radiologists (95% CI)	Advantage
Normal	1.0 (95% CI: 1.000–1.000)	0.991 (0.971–1.000)	AI
Fibrosis	0.950 (95% CI: 0.896–1.000)	0.900 (95% CI: 0.818–0.982)	AI
Heart shadow enlargement	0.991 (95% CI: 0.970–1.000)	0.980 (95% CI: 0.948–1.000)	AI
Mass	1.0 (95% CI: 1.000–1.000)	0.951 (95% CI: 0.896–1.000)	AI
Pleural effusion	0.993 (95% CI: 0.979–1.000))	0.949 (95% CI: 0.886–1.000)	AI
Pulmonary consolidation	0.982 (95% CI: 0.951–1.000)	0.904 (95% CI: 0.816–0.992))	AI
Aortic calcification	0.981 (95% CI: 0.953–1.000)	0.993 (95% CI: 0.978–1.000)	Radiologists
Calcification	0.915 (95% CI: 0.832–0.998)	0.933 (95% CI: 0.812–1.000)	Radiologists
Cavity	0.847 (95% CI: 0.742–0.952)	0.963 (95% CI: 0.906–1.000)	Radiologists
Nodule	0.881 (95% CI: 0.786–0.976)	0.923 (95% CI: 0.840–1.000)	Radiologists
Pleural thickening	0.895 (95% CI: 0.806–0.984)	0.957 (95% CI: 0.909–1.000)	Radiologists
Rib fracture	0.980 (95% CI: 0.937–1.000)	0.987 (95% CI: 0.958–1.000)	Radiologists
Subphrenic free air	1.0	1.0	No difference
Pneumothorax	1.0	1.0	No difference

Non-parametric bootstrapping was used to estimate the variability around each of the performance measures; 10,000 bootstrap replicates from the validation set were drawn, and each performance measure was calculated for the algorithm and the radiologists on these same 10,000 bootstrap replicates. This produced a distribution for each estimate, and the 95% confidence intervals (2.5th and 97.5th percentiles) are reported

*AUC* area under the receiver operating characteristic curve, *CI* confidence interval

### Significance of AI Assistance in Diagnosing Different Signs

The radiologists performed as well with AI as without AI for 2 signs, performed better with AI than without AI for 6 signs, and performed poorer with AI than without AI for the other 6 signs. The radiologists achieved AUCs of 1.0 (95% CI: 1.000–1.000), 0.950 (95% CI: 0.896–1.000), 0.991 (95% CI: 0.970–1.000), 1.0 (95% CI: 1.000–1.000), 0.993 (95% CI: 0.979–1.000), and 0.982 (95% CI: 0.951–1.000) with AI in normal, fibrosis, heart shadow enlargement, mass, pleural effusion, and pulmonary consolidation recognition, which were higher than the radiologists’ AUCs of 0.991 (0.971–1.000), 0.900 (95% CI: 0.818–0.982), 0.980 (95% CI: 0.948–1.000), 0.951 (95% CI: 0.896–1.000), 0.949 (95% CI: 0.886–1.000), and 0.904 (95% CI: 0.816–0.992) without AI. The radiologists achieved higher AUCs without AI in aortic calcification, calcification, cavity, nodule, pleural thickening, and rib fracture recognition, with AUCs of 0.993 (95% CI: 0.978–1.000), 0.933 (95% CI: 0.812–1.000), and 0.963 (95% CI: 0.906–1.000), 0.923 (95% CI: 0.840–1.000), 0.957 (95% CI: 0.909–1.000), and 0.987 (95% CI: 0.958–1.000), respectively, compared with AUCs of 0.981 (95% CI: 0.953–1.000), 0.915 (95% CI: 0.832–0.998), 0.847 (95% CI: 0.742–0.952), 0.881 (95% CI: 0.786–0.976), 0.895 (95% CI: 0.806–0.984), and 0.980 (95% CI: 0.937–1.000), respectively, with AI. There was no significant difference in the AUC (AUC = 1.0) for the other two signs, i.e., pneumothorax and subphrenic free air.

Using the relabeling procedure, the radiologists’ performance improved for 6 signs and worsened for 6 signs. The AI performed significantly worse than the radiologists in aortic calcification, calcification, cavity, nodule, pleural thickening, and rib fracture recognition, and the prevalence of all of these signs except nodules was low in the original training set. In pneumothorax and subphrenic free air recognition, however, the AI performed as well as the radiologists even though the prevalence of these signs in the original training set was low. In normal, fibrosis, heart shadow enlargement, mass, pleural effusion, and pulmonary consolidation recognition, the AI performed better than the radiologists.

## Discussion

This competition demonstrated the value of AI in detecting and localizing many pathologies in chest radiographs by simulating the real work situations of radiologists. The 111 radiologists participating in the competition independently completed 100 judgement tasks without AI assistance in the first 50 cases and with AI assistance in the last 50 cases. After the competition, the average scores of the participating radiologists were compared. We found that the average scores were significantly higher with than without AI assistance (*P* < 0.001). The radiologists were classified according to seniority, determined by years of experience. The average scores of intermediate and senior doctors were significantly higher with than without AI assistance (*P* < 0.05), while the average scores of junior doctors were similar with and without AI assistance (*P* > 0.05). Studies in the literature have illustrated the potential utility of AI models in improving the work efficiency and diagnostic accuracy of radiologists [12–15]. The present study shows that when radiologists are provided with the assistance of AI, their ability to detect disease can be significantly improved, thus diminishing diagnostic errors. However, there was no significant difference in the average scores of the junior doctors with and without AI assistance. There may be many reasons for this situation. The gold standard in our cases were the CT images in the same period. We inferred that this due to the false positives or false negatives generated by the AI [16], which are more difficult for junior doctors to eliminate. Less qualified doctors have more practical experience but lack diagnostic capacity and thus require higher-level doctors to review their reports based on day-to-day experience [17]. Secondly, it may be due to the relatively few X-rays used in the study, and subsequent studies need more cases to improve statistical ability. Lastly, it may also be due to individual differences in the group of junior radiologists participating in the competition, and more radiologists should be included to study this issue in the future. These speculations will be further verified in the future.

A chest X-ray screening system that automatically detects lung abnormalities can provide tremendous utility in countries where health care resources are constrained [2]. Furthermore, even experienced radiologists are still subject to human limitations, including fatigue, perceptual biases, and cognitive biases, all of which lead to errors [18–20]. Previous studies have shown that it is possible to reduce perception errors and prejudice by providing radiologists with feedback on the presence and location of abnormalities on X-ray images [21]. This situation is very suitable for our proposed algorithm.

At present, the main method of diagnosis based on chest X-ray images is to rely on radiologists to retrieve the images and compare them with the patient’s medical history [22]. The manual reading and large workloads significantly increase the job-related pressure of radiologists in examinations, leading to long-term, high-load, and high-tension work conditions for radiologists [23]. Such long-term work increases the fatigue of radiologists and increases the odds of missing the diagnosis of small lesions [24]. AI can accurately identify lesions and reduce the rate of missed diagnosis, but sometimes, it produces false positive and false negative results and gives incorrect advice to the doctors. Therefore, radiologists are required to review the lesions to improve the accuracy of the image diagnosis.

This competition simulates the daily work mode by comparing the first 50 radiographs assessed without AI assistance and the latter 50 radiographs assessed with AI assistance, confirming that the time for radiologists to assess images with AI assistance is shortened. In our competition, the average radiologist experienced a relative reduction in the misinterpretation rate of 3.01% and in the time required of 1353 s. The significant improvements in diagnostic accuracy that we observed in this competition showed that deep learning methods are a mechanism by which substantial improvements to patient care can be provided in radiology [25]. The shorter time required for all radiologists to assess the second 50 cases and the improvements in diagnostic accuracy show that AI assistance can enhance the efficiency and effectiveness of doctors.

Using AI to mark lesions and locations, the radiologists’ diagnostic effectiveness improved for six signs but decreased for six others. The competition demonstrated that the algorithm performed worse than radiologists in terms of recognizing aortic calcification, calcification, cavities, nodules, pleural thickening, and rib fractures. We infer that the reason for these results may be that in the original training set, the prevalence of all of these signs, except nodules, was low. However, in terms of pneumothorax and free air under the diaphragm, even if the prevalence of labels in the original training set is low, the effect of this algorithm is equivalent to that of radiologists. In terms of normal, fibrosis, heart shadow enlargement, mass, pleural effusion and lung consolidation recognition, the performance of this algorithm is better than that of radiologists. In fact, our research findings have shown that radiologists can efficiently diagnose 14 signs regardless of whether AI is used or not. The benefits of AI for radiologists are greatly improved diagnostic efficiency and stability. In addition, among the 14 individual signs, AI contributes to normal, fibrosis, enlargement of the heart shadow, masses, pleural effusion, and lung consolidation. The other 6 signs that do not benefit from AI are aortic calcification, calcification, cavities, nodules, pleural thickening, and rib fractures. The diagnostic efficiency of radiologists is also very high, and patients can benefit from the combination of radiologists and AI. The AI has lower sensitivity in detecting the three signs of rib fractures, nodules, and cavities. Radiologists are more cautious about these three signs, resulting in better results. This makes it difficult for AI to provide much help beyond the performance of individual radiologists. In future research, we will continue to optimize AI models to achieve higher performance. For the three signs of calcification, pleural thickening, and aortic calcification, it is easier to diagnose clinically, so radiologists have good results. We have added these explains in the discussion section.

Limitations of this study include some potential biases, which may cause the results of the radiologist evaluation and algorithm performance in the competition to be more conservative than in reality. First, the radiologists and algorithm only had access to frontal radiographs during reading, and it has been shown that up to 15% of accurate diagnoses require the lateral view [26]. The lack of a side view in the dataset may limit the detection of certain clinical findings, such as subtle pleural effusions that cannot be detected only on the frontal view. Future work may consider the use of lateral views when applicable in diagnosis and algorithm development. Second, the reference standard was determined by the consensus of thoracic radiologists on cross-sectional CT images. Histopathological examination is an ideal reference standard, but it is difficult or impossible to obtain pathological specimens in some disease states. Comparison with the pathological gold standard in all cases is beyond the scope and purpose of this study. Therefore, the goal was to use a forward-looking approach based on the interpretations of panels of experts to evaluate the performance of a deep learning algorithm in X-ray diagnostic tasks.

There are also several additional limitations that should be considered when interpreting our results. The main limitation of this study is that the dataset used for the competition was from a single institution; in our future work, we plan to address the generalizability of the algorithm to datasets from multiple institutions [27]. Additionally, the experimental design used to assess radiologist performance in this work does not replicate the clinical environment, so the radiologist performance scores presented in this study may not exactly reflect the true performance in a more realistic setting. Specifically, disagreement in chest radiograph interpretation between clinical radiologists has been well described and would not always be interpreted as an error in clinical practice. In that way, the labeling task performed by the radiologists in this study differs from routine clinical interpretation because in this work, all relevant findings in each image were labeled as present regardless of the potential clinical significance. Finally, comparison of the primary performance metric in this study required estimation of the ROC curve for radiologists. To evaluate the algorithm based on the results obtained, it is important to estimate how the predictive model will perform in practice based on several performance metrics. While we assumed that the specificity and sensitivity were balanced, allowing for a better fit, we acknowledge that this is not a perfect comparison, and for this reason, we also provided a comprehensive view of the algorithm performance metrics (S1).

## Conclusion

Through this competition, we showed that AI demonstrated comparable performance in assisting radiologists in detecting multiple chest abnormalities on frontal chest X-rays. Artificial intelligence methods can help to increase both the accuracy of the diagnoses and the efficiency of radiologists. Further studies are necessary to determine the feasibility of these outcomes in a prospective clinical setting.

### Supplementary Information

Below is the link to the electronic supplementary material.

Supplementary file1 (DOCX 14 KB)

## Data Availability

If the requirement is reasonable, any original data of this study can be obtained from the authors.

## Footnotes

1 *https://pytorch.org/docs/stable/index.html
