# Supplementary material for: Deep Learning for Chest X-ray Diagnosis: Competition Between Radiologists with or Without Artificial Intelligence Assistance
Source: J Imaging Inform Med. 2024 Feb 8;37(3):922–34. doi: 10.1007/s10278-024-00990-6 (PMC11169143; doi:10.1007/s10278-024-00990-6)
Supplement: Supplementary file 1 — Supplementary file1 (DOCX 14 KB) [file 10278_2024_990_MOESM1_ESM.docx]

**Supplement 1.** Performance of the Fast-RCNN model in a private dataset

We built a large private dataset (> 3000), and the sensitivity of the main signs is shown as follows:

| Algorithm function | Sensitivity (number of false positives per test) |
| --- | --- |
| 1 aortic calcification | 95% (0.17) |
| 2 heart shadow enlargement | 97% (0.2) |
| 3 Pneumothorax | 85% (0.2) |
| 4 pleural effusion | 82% (0.05) |
| 5 rib fracture | 68% (0.2) |
| 6 subphrenic free air | 80% (0.03) |
| 7 pleural thickening | 92% (0.2) |
| 8 pulmonary consolidation | 84% (0.2) |
| 9 mass | 87% (0.2) |
| 10 cavity | 84% (0.2) |
| 11 normal | 99% (0.2) |
| 12 nodule | 82% (0.2) |
| 13 fibrosis | 65% (0.2) |
| 14 calcification | 89% (0.2) |
